# Supplementary material for: Strong improvement of the transport characteristics of YBa2Cu3O7−x grain boundaries using ionic liquid gating
Source: Sci Rep. 2018 Dec 7;8:17703. doi: 10.1038/s41598-018-36166-w (PMC6286366; doi:10.1038/s41598-018-36166-w)
Supplement: Supplementary file 1 — Supplementary Information [file 41598_2018_36166_MOESM1_ESM.pdf]

Strong improvement of the transport characteristics of $YBa_2Cu_3O_{7-x}$ grain boundaries using ionic liquid gating

A. Fête[1,*] and C. Senatore[1]

[1]Department of Quantum Matter Physics (DQMP), University of Geneva, Geneva, Switzerland
[*]alexandre.fete@unige.ch

T_c vs J_c evolution of the GB-free part of our channels

In this section, we present data recorded using taps $V_{1,2}$ (GB-free regions). In this case, we used a $1\,\mu\mathrm{V\,cm}^{-1}$ criterion to define J_c and $R = 0$ to define T_c. Using the fact that for films thinner than their London penetration depth J_c can be linked to the superfluid density[1], we draw in Fig. S1 a Uemura plot. Repeating the procedure presented in[2], we compare our data with the ones from[3,4], acquired on thin films and bulks. Clearly, our results agree very well with the previously published literature on thin films. Actually, the agreement is even better than what we previously published[2]. This can be due to our optimized growth conditions and to the slightly thicker films investigated here. Indeed, these parameters can modify the superconducting transition width and hence our estimation of T_c.

As mentioned in the main text, the agreement observed in Fig. S1 is a good indication that IL liquid gating is doping our structures in an homogenous way. Otherwise, first, the power law linking T_c and $1/\lambda^2$ would be very different from what has been published on chemically doped films. Second, in a non-homogenous doping scenario, increasing the film thickness from 5 to 10uc, would lead to a steady deviation of the power law behavior. More details on this procedure can be found in[2]

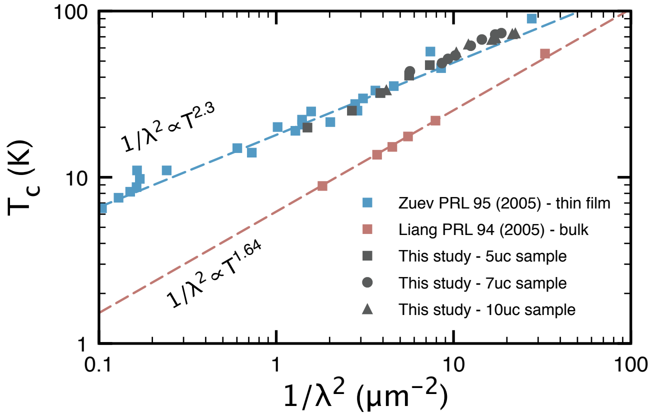

Figure S1. Uemura plot of the GB-free regions of our samples (taps $V_{1,2}$). Reference data from chemically doped bulks[3] and thin films[4] are shown for comparison.

References

1. Talantsev, E. F. & Tallon, J. L. Universal self-field critical current for thin-film superconductors. *Nat. Commun.* **6**, 7820 (2015). DOI 10.1038/ncomms8820.
2. Fete, A., Rossi, L., Augieri, A. & Senatore, C. Ionic liquid gating of ultra-thin $YBa_2Cu_3O_{7-x}$ films. *Appl. Phys. Lett.* **109**, 192601 (2016).
3. Liang, R., Bonn, D. A., Hardy, W. N. & Broun, D. Lower Critical Field and Superfluid Density of Highly Underdoped $YBa_2Cu_3O_{6+x}$ Single Crystals. *Phys. Rev. Lett.* **94**, 117001 (2005).
4. Zuev, Y., Seog Kim, M. & Lemberger, T. R. Correlation between Superfluid Density and TC of Underdoped $YBa_2Cu_3O_x$ Near the Superconductor-Insulator Transition. *Phys. Rev. Lett.* **95**, 137002 (2005).
